# Integrating Suicide Risk Screening into Pediatric Ambulatory Subspecialty Care

**DOI:** 10.1097/pq9.0000000000000310

**Published:** 2020-06-08

**Authors:** Becky H. Lois, Tamaki H. Urban, Christina Wong, Erin Collins, Lara Brodzinsky, Mary Ann Harris, Hayley Adkisson, Monique Armstrong, Jeanmarie Pontieri, Diana Delgado, Jeremiah Levine, K. Ron-Li Liaw

**Affiliations:** From the * Department of Child and Adolescent Psychiatry, NYU Langone Health, New York, N.Y.; † Department of Psychiatry and Behavioral Sciences, Northwestern University Feinberg School of Medicine, Chicago, Ill.; ‡ Medical Center Information Technology, NYU Langone Health, New York, N.Y.; § Department of Social Work, NYU Langone Health, New York, N.Y.; ¶ Department of Pediatrics, NYU Langone Health, New York, N.Y.

## Abstract

**Background:**

Suicide risk screening is recommended in pediatric care. To date, no previous studies illustrate the implementation of suicide risk screening in pediatric subspecialty care, even though chronic medical conditions are associated with a higher risk of suicide.

**Methods:**

A large multidivision pediatric ambulatory clinic implemented annual suicide risk screening. Patients ages 9–21 years participated in suicide risk screening using the Ask Suicide-Screening Questions during the project. A multidisciplinary team employed quality improvement methods and survey-research design methods to evaluate the feasibility and acceptability of the screening process for patients, families, and medical providers.

**Results:**

During the quality improvement project period, 1,934 patients were offered screening; 1,301 (67.3%) patients completed screening; 82 patients (6.3% of 1,301 patients) screened positive. The monthly compliance rate held steady at 86% following several Plan-Do-Study-Act cycles of improvement. The survey results demonstrate that providers rated the suicide risk screening process positively; however, a subset of providers indicated that the screening process was out of their scope of practice or impeded their workflow.

**Conclusions:**

Suicide risk screening is feasible in pediatric specialty care and can identify at-risk patients. Continued efforts are needed to standardize suicide risk screening practices. Future directions include identifying factors associated with suicide risk in patients in pediatric subspecialty care settings.

## INTRODUCTION

Suicide is the second leading cause of death for youth ages 10–24.^1^ Seven percent of high school youth report that they have made at least 1 suicide attempt within the past 12 months.^1^ Children with chronic medical conditions are twice as likely to present with suicide risk than healthy controls (odds ratio range: 1.89–2.5).^2–7^

Seventy-eight percent of children who die by suicide have had contact with a healthcare provider within a year before their death, and 38% of young people have had contact within 4 weeks of dying.^8,9^ Early identification and treatment of youth at elevated risk for suicide is a key suicide prevention strategy, yet healthcare providers often do not recognize high-risk patients. Unfortunately, patients infrequently discuss suicidal thoughts and plans unless asked directly. The Joint Commission^10^ issued a Sentinel Event alert for hospital settings recommending annual suicide risk screening for all patients. The American Academy of Pediatrics also recommends suicide risk screening.^11^

Previous studies have indicated the feasibility and acceptability of screening programs in diverse pediatric healthcare settings.^12–14^ However, building screening into a medical appointment workflow is challenging and has led to inconsistent screening practices.^15^ Brahmbhatt et al^16^ recently published standardized clinical guidelines for the implementation of suicide risk screening in pediatric emergency departments and inpatient units. However, no studies to date have illustrated the application of suicide risk screening across pediatric subspecialty care.

A large multidivision pediatric ambulatory practice implemented annual suicide risk screening to (1) build a systemized, integrated assessment, and response workflow via a quality improvement (QI) framework and (2) shift the culture of a subspecialty medical clinic from a purely medical model to a more holistic model of care. This article aims to describe the implementation, feasibility, and acceptability of suicide risk screening in a pediatric ambulatory subspecialty setting, as evidenced by compliance with screening practices and prevalence of positive screens and their impact on workflow.

## METHODS

### Sample

This project was implemented with patients ages 9 and up in a large urban ambulatory subspecialty clinic within a children’s hospital in the Northeast. The clinic includes 7 subspecialty services—endocrinology, gastroenterology, pulmonology, cardiology, adolescent medicine, infectious disease, and nephrology, with 30,000 patient encounters per year. The clinic includes an embedded Integrated Behavioral Health (IBH) team, consisting of psychologists, social workers, and child life specialists. All providers use the Epic electronic medical record (EMR, Epic Systems, Verona, Wis.).

### Intervention

**Fig. 1.**
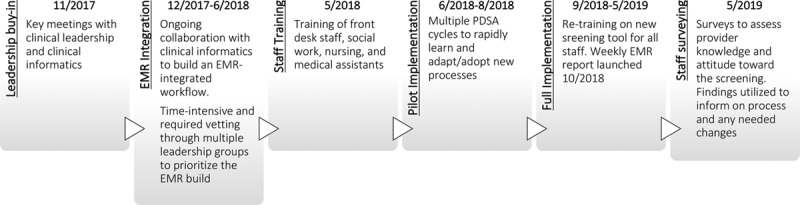
Timeline of screening implementation.

See below for a listing of the phases of suicide risk screening implementation; Figure 1 includes a timeline summary.

#### Establishing Leadership Support

**Fig. 2.**
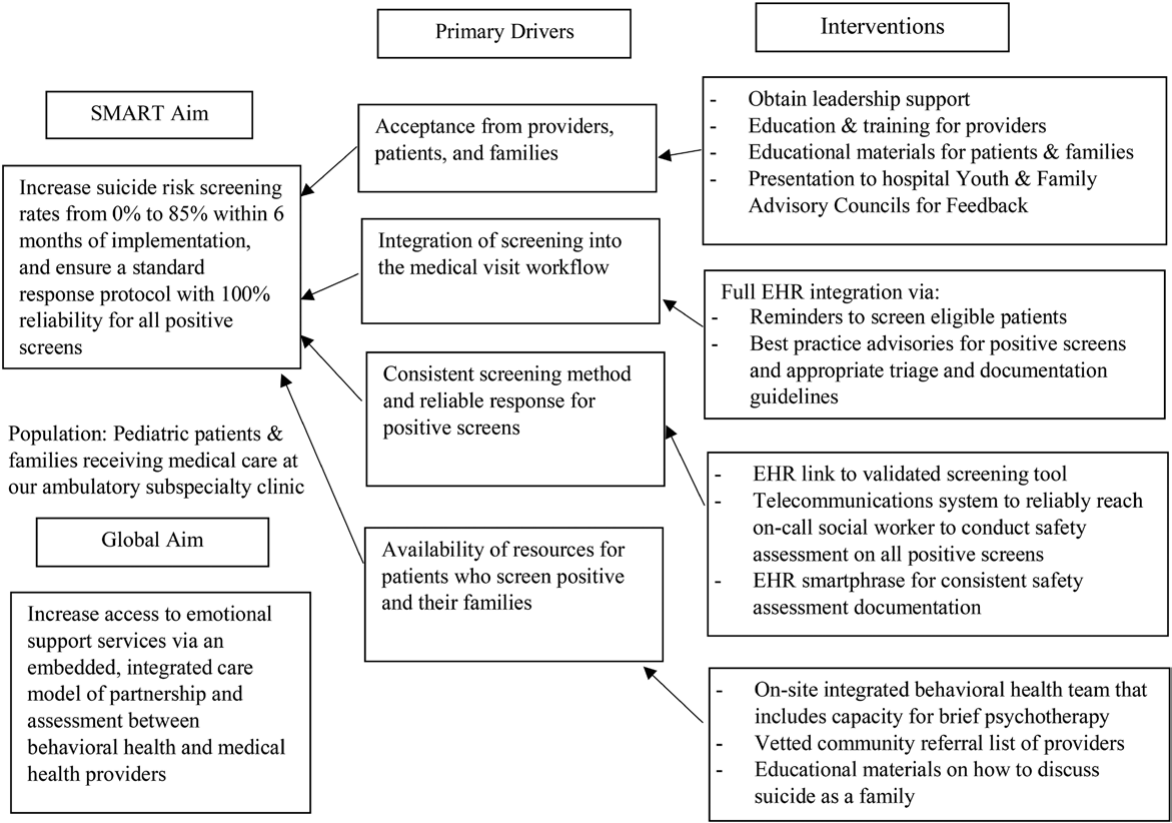
Key driver diagram of screening project.

The project leaders (a pediatric psychologist and child psychiatrist) partnered with clinic leadership and a medical informatics team. This interdisciplinary QI team discussed the rationale for screening in a medical setting and resources required for clinical workflow and EMR integration. The team created a key driver diagram to identify interventions in line with the project’s aims (Fig. 2).

#### Selecting a Screening Tool

Our clinic adopted a clinical pathway model^16^ that recommends using the Ask Suicide-Screening Questions (ASQ; see Measures section).^17^ Social workers utilized the ASQ Brief Suicide Safety Assessment (ASQ-BSSA) for second-tier assessment and further triage of positive screens.

#### Determining the Workflow

Given the clinic’s many specialty divisions with different workflows, it was challenging to establish a standard, efficient workflow. Front desk staff provided an informational handout about the screening initiative to patients and families. Medical assistants (MAs), who triage every patient, were identified as the most appropriate screening administrators. The MAs conducted the screening with the following process:

1. Introduce the suicide risk screening initiative at the end of medical triage.
2. Request permission only from parents of children ages 9–12, per the hospital’s requirement (all other patients and families could decline to participate, but were not explicitly asked for permission to screen).
3. Ask the parent to step out of the room for patient privacy if the patient and parent were comfortable.
4. Give the patient a paper copy of the ASQ to complete.
5. Collect the screen, review and enter results into the EMR, and communicate findings to the patient and parent. Regarding the decision to have patients to complete the screen on paper, MAs conducted medical triage in a shared space that often did not allow for confidential conversations.

If a patient answered in the affirmative to any questions, the response was considered a positive screen. If a patient answered yes to questions 1–4 only, the screen was classified as a nonacute positive, and the MA would call the on-call social worker to conduct the ASQ-BSSA,18 in line with tier 2 of the suicide screening pathway.^15^ If a patient answered yes to Question 5 (“Are you having thoughts about killing yourself right now?”), the screen was classified as an acute positive. It required the initiation of one-to-one care by staff to ensure patient safety.^19^ These patients required full safety precautions and comprehensive psychiatric evaluation (tier 3 of the pathway). If a patient refused to answer an item or the MA had a concern about a patient despite his/her response to the screen, the MA was instructed to call the on-call social worker for further assessment.

**Fig. 3.**
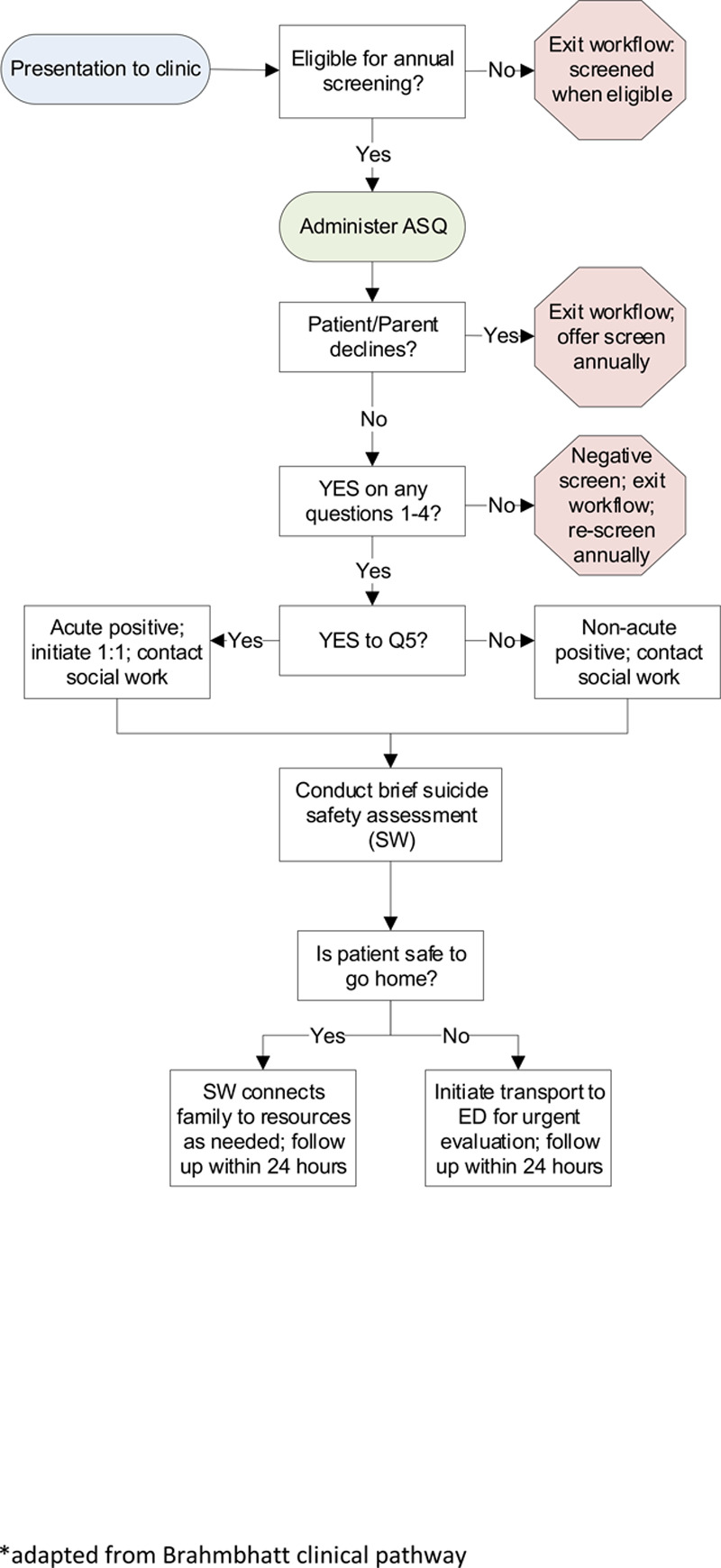
Process map illustrating the screening workflow.

The clinic has 3 full-time social workers who conducted brief suicide safety assessments for any positive screens. Two clinical psychologists were available for on-site consultation as needed. See Figure 3 for a detailed process map depicting the screening workflow.

#### Building the Infrastructure

To ensure that the screening process was efficient and reliable, the QI team needed to align with the health system’s Medical Center Information Technology (MCIT) team. MCIT built an EMR workflow for the screening process which included: an alert to cue staff that a patient was due for an annual suicide risk screen; a flow sheet to chart each patient’s responses; and alerts for the administrator to classify screens as “positive” and the subsequent actions to ensure safety. The MCIT team created a weekly report, which allowed the QI team to track compliance with the screening process and deploy targeted change strategies week-to-week to improve response reliability.

#### Training the Team

The IBH team psychologists developed and conducted 1-hour, discipline-specific training sessions regarding the screening protocol for social work, nursing, and MAs. The IBH team held separate 1-hour training for front desk staff and medical providers (MPs) consisting of physicians and nurse practitioners. The psychologists have also provided ongoing weekly education and support for the MAs to help them process their emotional experiences and build skills.

#### Pilot Implementation: Plan-Do-Study-Act Cycles

Implementation occurred via a QI framework, with several Plan-Do-Study-Act (PDSA) cycles designed to rapidly learn from small tests of change and adapt/adopt processes for subsequent cycles.^18^ The clinic implemented the first PDSA cycle tests, with each nurse administering 1 screen on 1 patient per day for 1 week. Nurses were chosen as the first pilot implementation group because they already had screening administration competencies from previous work in inpatient units. For the first month of implementation, the team held huddles each morning to ensure fidelity to the screening protocol and each afternoon to debrief on the day’s learning and make any necessary changes ahead of the next day. Subsequent PDSA cycles were implemented once the task of screening administration shifted to the MAs.

#### Full Implementation

**Fig. 4.**
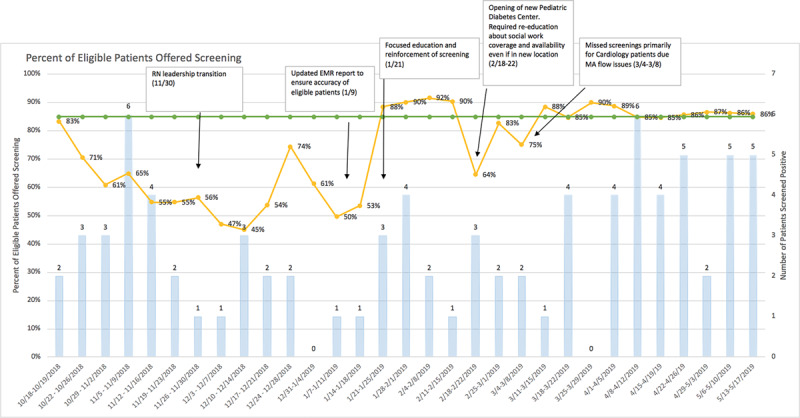
Run chart depicting the reliability of the screening process over time.

After the implementation of the PDSA cycles, the QI team prioritized EMR optimization to maximize efficiency in the workflow. Upon completion of the EMR build, the project shifted to full implementation. See Figure 4 for the run chart depicting screening reliability and additional PDSA cycles.

### Measures

#### Suicide Risk

The clinic utilized the ASQ^17^ to assess suicide risk in patients. The ASQ was developed to screen for suicide risk in children and youth in medical settings. The tool has strong psychometric properties, typically takes 20 seconds to administer, and has clear triage guidelines for positive responses.^17^

#### Feasibility and Acceptability of Suicide Risk Screening for Patient, Family, and Provider

The QI team surveyed patients and families via presentations at the hospital’s Family and Youth Advisory Councils. The QI team presented the screening project to each Council separately to ask specific questions about how patients and families would want the screen to be presented and how they would like to receive results. A facilitator guided the discussion and took verbatim notes to share with the QI team.

Additionally, the QI team created and implemented a 10-question survey (Table 1) of the MPs and MAs. The surveys consisted of a 5-point Likert scale, yes/no questions, and free description questions related to attitude, knowledge, comfortability, emotional burden, and systematic issues that they were facing regarding the screening. The QI team elected to survey MPs despite their lack of direct involvement in the screening process, given the importance of their role for patients and families at the clinic and the need for their support as critical stakeholders. MAs were asked the same questions as the MPs, with a few additional questions pertinent to their role in the process.

**Table 1.**
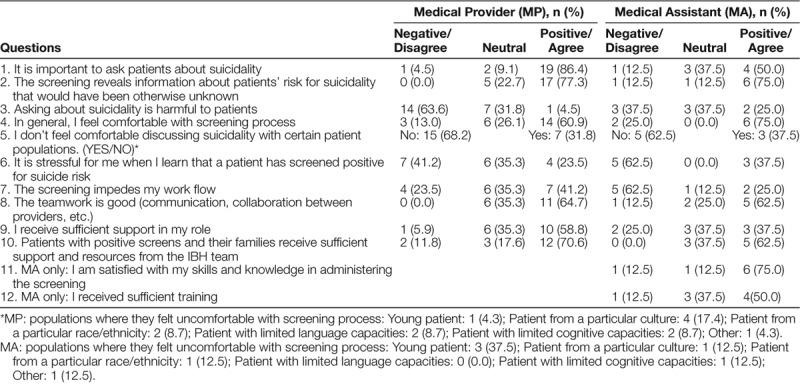
Medical Provider and Medical Assistant Survey Results

### Data Collection and Analysis

We collected data through standard clinical practice. Inclusion criteria for this review are patients: (1) who received care and were offered suicide risk screening using the ASQ at our clinic from October 2018 to May 2019 and (2) 9–21 years of age. Additionally, patients ineligible for suicide risk screening as determined by the MAs (eg, outside the age range, developmental disabilities that prevent understanding of screening questions, nonverbal) were excluded. The QI team created a run chart to track the weekly compliance rates (Fig. 4). This project was reviewed and classified as QI and not human subject research by the hospital’s Institutional Review Board.

## RESULTS

### Demographics

Table 2 shows the characteristics of 1,934 patients whom the MAs offered screening. The sample was 53.6% white and 50.2% male with an average age of 13.7.

**Table 2.**
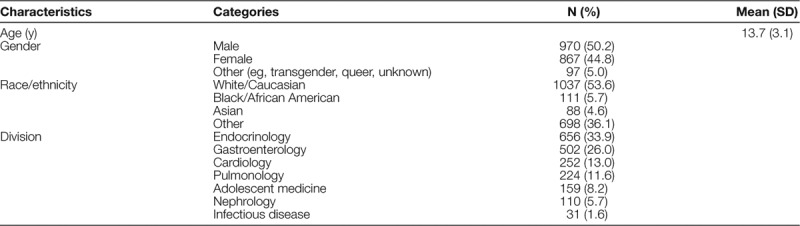
Characteristics of Sample of Eligible Participants (N = 1,934)

### Screening Data

Of 1,934 patients who were offered screening, 67.3% (n = 1,301) completed screening and 32.7% (n = 633) elected to not complete screening. Of patients who completed screening, 6.3% (82 of 1,301 patients) screened positive. All positive screens resulted in a response by on-site social workers for immediate safety assessment and resource connection. Of patients who screened positive, 2.4% (2 of 82 patients) were acutely positive; both patients required transport to the hospital’s emergency department for urgent evaluation. Neither patient was subsequently psychiatrically hospitalized. Of the nonacutely positive patients, 51.2% (41 out of 80 patients) were already connected to community care; 10.0% (n = 8) elected to be seen at our clinic by our psychology team, and 38.8% (n = 31) requested and received a connection to resources in their community.

### Screening Compliance

During the eight-month project period, total screening compliance averaged 73.5%. The clinic’s nurse manager role transitioned 1 month aftert pilot implementation, which resulted in several PDSA cycles for EMR optimization and staff retraining over several months. These rapid improvement cycles resulted in an average compliance rate of 85% during the last 12 weeks, which met the goal set at the outset of the pilot.

## QUALITATIVE SURVEYS AND FEEDBACK: PROVIDERS (MPS, MAS) AND PATIENTS AND FAMILIES

### Providers

In total, 74% of MPs (n = 23) and 100% of MAs (n = 8) completed the survey (Table 1). For ease of interpretation, Likert data were collapsed into positive (somewhat/strongly agree), neutral, and negative (somewhat/strongly disagree) responses.

A majority of MPs (59.1%) and MAs (75%) felt comfortable with the screening process. Most MPs (77.3%) and MAs (75.0%) indicated an awareness that the screening yielded valuable information about suicide risk. Conversely, 17.4% of MPs reported discomfort with patients from a particular culture because they felt “(it’s) not area of my practice” or “I’m not qualified.” A cohort of MAs (37.5%) reported discomfort with young patients ages 9–11 during the screening process because “parents get defensive” (eg, declining screening, expressing a fear that screening can increase or stimulate suicidality in their children).

Most MPs (41.2%) and MAs (62.5%) denied significant feelings of stress when a patient screened positive. Although 41.2% of MPs agreed that the screening process impeded their workflow, only 25.0% of MA agreed.

A majority of MPs (64.7%) and MAs (62.5%) reported positively regarding the level of teamwork (ie, collaboration across MPs, MAs, and social workers). MPs reported that they received sufficient support in their role (58.8%) and support from the QI team regarding implementation (70.6%). The majority of MAs indicated either neutral or positive responses to a question asking about sufficient training (87.5%); they indicated neutral or negative responses regarding adequate support (62.5%) from their bosses or supervisors.

### Patients and Families

Parent members of the Family Advisory Council (18 parents present; 10 provided direct feedback) shared thoughts on the complexities of sharing screening results with their doctors and messaging to families when children screened positive: “I totally understand why someone would want their doctor asking their questions. But I feel like I have a very specific relationship with my doctor. If I was having those kinds of thoughts, I wouldn’t want that affecting how they handle my care.” “I always liked when they gave the margin of error. Like in 95% of cases there’s nothing to worry about. This is step 1 of many steps.” Pediatric patients on the Youth Advisory Council (7 patients present; all provided direct feedback) appreciated the direct nature of the questions and preferred that the screen be completed on paper or in a modality chosen by the patient: “it’s good that they’re direct so they aren’t misinterpreted.” “Can you give patients the choice of paper or in person? Some people don’t like “tests” or second guess their answers when writing.”

## DISCUSSION

This QI project aimed to determine the feasibility and acceptability of the screening process. Overall, findings indicate that screening of suicide risk in a pediatric subspecialty setting is feasible, acceptable, and essential. Of the 1,301 patients screened, the positive screen rate was 6.3%, with an acute positive rate of 0.2%; these proportions indicate a low burden on the clinic system. The workflow identified eligible patients who were subsequently offered screening during their medical visits, with any positive screens leading to a 100% response from on-site social workers. The QI team has held steady with compliance rates at or above 85% for the past 3 months despite the busy nature of a multidivision ambulatory practice.

Overall, patients, families, MPs, and MAs positively rated the importance of suicide risk screening. Providers across disciplines agreed on several points, including that the screening reveals patients’ suicidal risk that would have been otherwise undetected, and that the screening is helpful to patients. Nevertheless, some of the MPs and MAs felt uncomfortable during screening or acknowledged stress upon positive screening. Some MPs also felt uncomfortable in the process of screening patients from a particular culture. The clinic includes a significant population of families who identify as Orthodox Jewish, and providers have anecdotally shared concerns about approaching this population for screening. Mental illness can stigmatize Orthodox Jewish individuals as well as their families,^20,^ which may discourage these families from speaking to mental health professionals^21^ and may, in turn, make asking these questions challenging. At a systemic level, more MPs reported impeded workflow due to screening than MAs. Additionally, surveys indicated a need for more support for MAs. The QI team has organized additional support for the MAs and all clinic staff to highlight their important work and the clinic’s commitment to their well-being.

Our sample had a relatively high decline rate (average rate of 32.7%). However, this rate is similar to those previously seen in an emergency department setting^11^ and still indicates that most parents found it acceptable to participate. MAs were required to obtain parental permission to screen children ages 9–12, per our hospital’s policy, which likely led to a higher proportion of families opting out of the screening in this age group. Given the positivity rates found in our sample as well as the Joint Commission’s recommendations for screening, we recommend that screening becomes standard of care, like other medical procedures conducted during triage like blood pressure and temperature. Additionally, the QI team is surveying additional clinic patients and families to understand their perspective on the screening process better. The QI team also plans to augment educational materials for families on the myth of iatrogenic risk of screening for suicide risk and to adapting these materials for wider cultural acceptability.

## LIMITATIONS

First, although we offered the screening to all eligible patients, their participation was voluntary, limiting the generalization to all patients in pediatric subspecialty care. Second, the screening process differed for patients 9–11, as parental assent was required. Patients and families who “opted in” to screening may have led to selection bias, and thus the sample may not be representative of the whole. Third, our data did not include information as to whether children declined the screening, or parents did. Furthermore, data did not include demographic information on patients who declined to participate, which precludes us from comparing any differences across groups. Not having this information makes it challenging to improve participation in screening, as it is unclear how to best target the issue without understanding any trends or points of comparison.

## CONCLUDING SUMMARY

Overall, universal screening canidentify patients at risk and improve quality, safety, and experience through a more holistic approach to healthcare. Key learning from this QI project included the importance of (1) providing the front desk with the informational flyers to families, as it decreased burden for MAs in discussing the screening and its purpose; (2) having strategies to manage issues of confidentiality (eg, paper screen vs. verbal report) because triage rooms held multiple patients; (3) automating the EMR system alerts for safety and reliability; (4) creating resource lists to ensure that families are connected to vetted community providers efficiently; (5) ongoing training and weekly support for all staff to debrief distress; and (6) ensuring the presence of on-site clinical social workers to conduct safety assessments and resource connection. Continued efforts are needed to reduce provider stress and discomfort with screening. Further studies should examine the outcomes of screening and their impact on future suicidal behavior.

## DISCLOSURE

The authors have no financial interest to declare in relation to the content of this article.

## ACKNOWLEDGMENTS

We thank our clinic’s medical assistant team for all of their hard work and efforts in keeping our patients safe: Jacqueline Ahia, Sherell Hubbard, Jessica Lindner, Angel Rodriguez, Jose Mercado, Nidia Velasquez, Roseline Famewo, Emeline Figueroa, Yajaira Hernandez, and Ladonice Bullock. We thank Morgan Pacheco, LCSW, for her efforts as a member of our senior social work team during the early stages of implementation, and Ellen Waggett for her review of the manuscript from the lens of a parent. Last, we thank all of our patients and families, from whom we learn every day.
